# Variants of *Escherichia coli* Subtilase Cytotoxin Subunits Show Differences in Complex Formation In Vitro

**DOI:** 10.3390/toxins11120703

**Published:** 2019-12-03

**Authors:** Maike Krause, Katharina Sessler, Anna Kaziales, Richard Grahl, Sabrina Noettger, Holger Barth, Herbert Schmidt

**Affiliations:** 1 Department of Food Microbiology and Hygiene, Institute of Food Science and Biotechnology, Garbenstraße 28, University of Hohenheim, 70599 Stuttgart, Germany; maike.krause@uni-hohenheim.de (M.K.); richard.grahl@gmx.de (R.G.); 2 Institute of Pharmacology and Toxicology, University of Ulm Medical Center, Albert-Einstein-Allee 11, 89081 Ulm, Germany; katharina.sessler@uni-ulm.de (K.S.); sabrina.noettger@uni-ulm.de (S.N.); holger.barth@uni-ulm.de (H.B.); 3 Center for Integrated Protein Science Munich, Department of Chemistry, Technical University of Munich, Lichtenbergstr. 4, 85748 Garching, Germany; anna.kaziales@tum.de

**Keywords:** subtilase cytotoxin, AB_5_ toxin, complex formation, STEC, analytical ultracentrifugation, isothermal titration calorimetry, flow cytometry

## Abstract

The subtilase cytotoxin (SubAB) of Shiga toxin-producing *Escherichia coli* (STEC) is a member of the AB_5_ toxin family. In the current study, we analyzed the formation of active homo- and hetero-complexes of SubAB variants in vitro to characterize the mode of assembly of the subunits. Recombinant SubA1-His, SubB1-His, SubA2-2-His, and SubB2-2-His subunits, and His-tag-free SubA2-2 were separately expressed, purified, and biochemically characterized by circular dichroism (CD) spectroscopy, size-exclusion chromatography (SEC), and analytical ultracentrifugation (aUC). To confirm their biological activity, cytotoxicity assays were performed with HeLa cells. The formation of AB_5_ complexes was investigated with aUC and isothermal titration calorimetry (ITC). Binding of SubAB2-2-His to HeLa cells was characterized with flow cytometry (FACS). Cytotoxicity experiments revealed that the analyzed recombinant subtilase subunits were biochemically functional and capable of intoxicating HeLa cells. Inhibition of cytotoxicity by Brefeldin A demonstrated that the cleavage is specific. All His-tagged subunits, as well as the non-tagged SubA2-2 subunit, showed the expected secondary structural compositions and oligomerization. Whereas SubAB1-His complexes could be reconstituted in solution, and revealed a *K_d_* value of 3.9 ± 0.8 μmol/L in the lower micromolar range, only transient interactions were observed for the subunits of SubAB2-2-His in solution, which did not result in any binding constant when analyzed with ITC. Additional studies on the binding characteristics of SubAB2-2-His on HeLa cells revealed that the formation of transient complexes improved binding to the target cells. Conclusively, we hypothesize that SubAB variants exhibit different characteristics in their binding behavior to their target cells.

## 1. Introduction

The genes encoding the subtilase cytotoxin (SubAB) are present in various Shiga toxin-producing *Escherichia coli* (STEC) strains [1,2,3]. SubAB was originally found and characterized in the *E. coli* O113:H21 strain 98NK2, which was isolated from a patient suffering from hemolytic uremic syndrome (HUS) [4,5]. Several virulence profiling studies showed that *subAB* genes are often present in sheep and wild ruminant STEC isolates. [3,6,7]. In addition, such STEC did not contain the locus of enterocyte effacement (LEE) [8,9,10]. To our knowledge, *subAB* genes are always detected in Shiga toxin (*stx*)-positive strains. Therefore, it was hypothesized that the pathogenic function of SubAB is linked to that of Stx [5,11]. However, animal studies gave first insights into the pathogenesis by intoxication with only SubAB [5,11,12,13]. For example, Seyahian et al. [13] showed that SubAB1 injection in sublethal doses causes severe damages in kidneys, hearts, and livers in rats, which can contribute to HUS pathogenesis. Comparable damages were already described for mice and were linked to HUS symptoms observed in humans [5,11]. Therefore, SubAB may be considered as a pathogenicity factor, which contributes to the virulence of STEC.

The genes encoding SubAB1 are located on the virulence plasmid pO113 [5]. In addition to the plasmid-located *subAB_1_* genes, the chromosomal variant genes *subAB_2-1_*, *subAB_2-2_*, and *subAB_2-3_* were described [5,6,14,15], and further *subAB* variant genes were suggested [16].

SubAB is composed of an enzymatically active A-subunit (SubA) and five B-subunits (SubB), the latter mediating the binding of the toxin to its target cells. Byres et al. [17] identified *N*-glycolylneuraminic acid (Neu5Gc), which is α2,3-linked to a glycan receptor, as a binding target of the B-pentamer. However, latest studies revealed that SubAB may be able to bind to a variety of glycans on the cell surface. In particular, *N*-glycans seem to be the main binding partners, and *O*-glycans play only a minor role in target cell recognition by SubAB [18]. Upon binding, the holo-toxin is retrogradely transported to the endoplasmic reticulum, where the A-subunit binds to the endoplasmic Hsp70 chaperone BiP/GRP78 [19]. SubA specifically cleaves BiP at the L–L motif between its ATPase and substrate-binding domain, causing an unfold protein (UPR) stress response that ultimately leads to apoptosis of the target cell [19,20].

The assembly of AB_5_ toxins seems to be a crucial step for their functionality. Some members of this family are released from the cell by specific secretion systems as holo-toxin complexes [21,22]. Thus, it was assumed that Stx and SubAB also form stable complexes before binding to their targets. However, Pellino et al. [23] showed that preassembly of Stx1 and Stx2 is not required for successful intoxication. They proposed that binding of the B-subunits to their receptor on the target cell could be a template for the formation of holo-toxin complexes. Funk et al. [24] demonstrated that the separately expressed A- and B-subunits of different SubAB variants are able to form functional homo- and hetero-complexes with high cytotoxic activity. Currently, it is not known whether SubAB subunits assemble in solution to form stable toxin complexes.

The aim of the current study was to analyze the capability of recombinant SubAB subunits to form homo- and hetero-complexes in vitro. Therefore, effective purification protocols were established, followed by analyzing the activity of the purified proteins by cytotoxicity assays on HeLa cells. Subsequently, the separately expressed and purified recombinant SubAB subunits were analyzed biochemically using far-ultraviolet (UV) circular dichroism (CD) spectroscopy and size-exclusion chromatography (SEC). The homo- and hetero-complex formations were characterized with analytical ultracentrifugation (aUC). If possible, dissociation constants *K_d_* were determined by isothermal titration calorimetry (ITC). In addition, we performed flow cytometry (FACS) analysis to further elucidate binding of SubAB complexes to target cells when no stable toxin complexes were detectable in solution.

## 2. Results

### 2.1. Purification of SubA and SubB Subunits

In order to study the association of different subtilase subunits, SubAB1 and the recently described variant SubAB2-2 were selected. These variants share an overall identity in their amino-acid sequences of 94.5% for the A-subunits and 92.4% for the B-subunits, but they have distinct genetic localizations in the STEC genomes. Since previous studies showed differences in CD_50_ values of these variants [24], we were interested to further characterize them in their ability to form active complexes.

The His-tagged subunits of SubAB1 and SubAB2-2 were expressed in *E. coli* C41 (DE3) cells as described elsewhere [24]. The purification protocols for these subunits established by Funk et al. [24] were improved by adding size-exclusion chromatography steps to allow the removal of imidazole and further impurities of the tagged subunits as described below.

In addition, the SubA2-2 subunit was cloned, expressed, and purified without His-tag. Therefore, the *subA_2_*_-2_ gene was amplified using a previously published expression vector for His-tagged SubA2-2 [24]. Subsequently, *subA_2_*_-2_ was cloned into a pET16b(+) expression vector to generate the expression plasmid pKR01. The correct insertion of the insert was confirmed by Sanger sequencing (see below). Auto-induction medium according to Studier [25] containing 0.2% (*w/v*) lactose was used for the expression of SubA2-2 with high reproducibility in *E. coli* C41 (DE3). This medium exploits the natural change in the bacterial metabolism when the carbon source changes from glucose to lactose, which automatically activates the T7-promoter on the pET16b(+) vector [25].

A two-column approach on an ÄKTA pure chromatography system was used for purification of SubA2-2 as described below in detail. Cell lysates containing SubA2-2 were loaded on a sulfopropyl (SP) Sepharose Fast Flow (SPff) column to capture the subunit. After elution with a linear salt gradient, SubA2-2 was further purified by size-exclusion chromatography using a Superdex75pg column.

**Figure 1:**
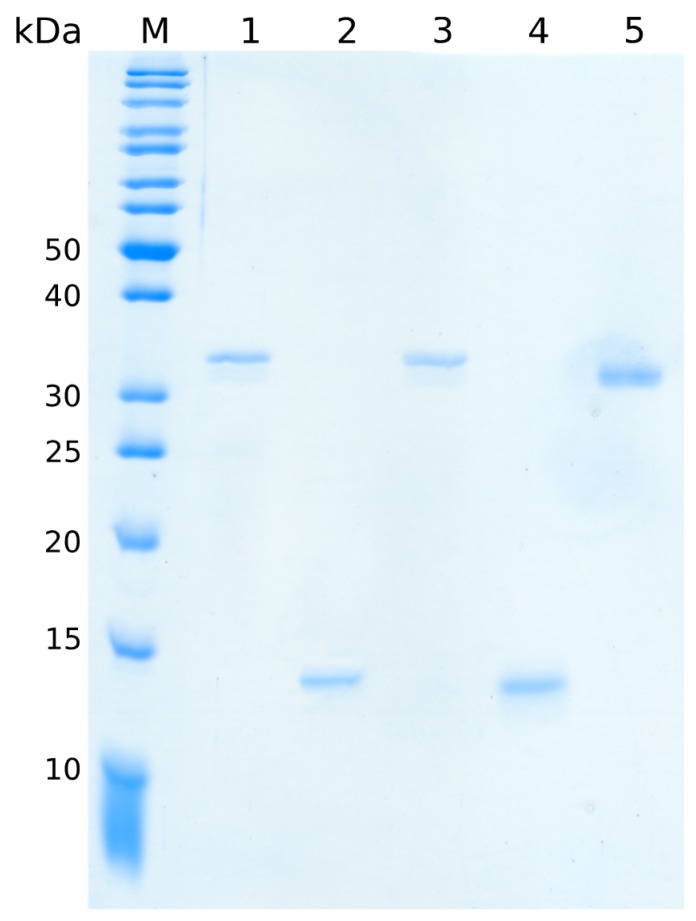
12.5% SDS-PAGE of separate subtilase cytotoxin (SubA and SubB) subunits. Lanes 1, 2, 3, 4, and 5 show SubA1-His, SubB1-His, SubA2-2-His, SubB2-2-His, and SubA2-2 bands, respectively. A total protein amount of 200 ng was applied to each lane. M represents the protein marker (PageRuler™ unstained protein ladder, Thermo Fisher Scientific, Waltham, MA, USA). The size of the relevant marker proteins is indicated on the left.

With the refinement of the purification protocol for SubAB1-His and SubAB2-2-His subunits and the newly developed expression and purification strategy for SubA2-2, all protein preparations revealed a purity of at least 95%. The result shown in Figure 1 visualizes representative samples of the purified proteins.

SubA1-His (lane 1) and SubA2-2-His (lane 3) appeared as bands of 33 kDa. SubB1-His (lane 2) and SubB2-2-His (lane 4) appeared as 14-kDa bands. These molecular weights corresponded to the theoretical molecular weight of the toxin subunits without the signal peptides at their amino termini. SubA2-2 (lane 5) appeared with approximately 32 kDa on the gel, suggesting that the signal peptide of 21 amino acids for the A-subunit was also cleaved off during expression.

### 2.2. Cytotoxicity Assay

**Figure 2:**
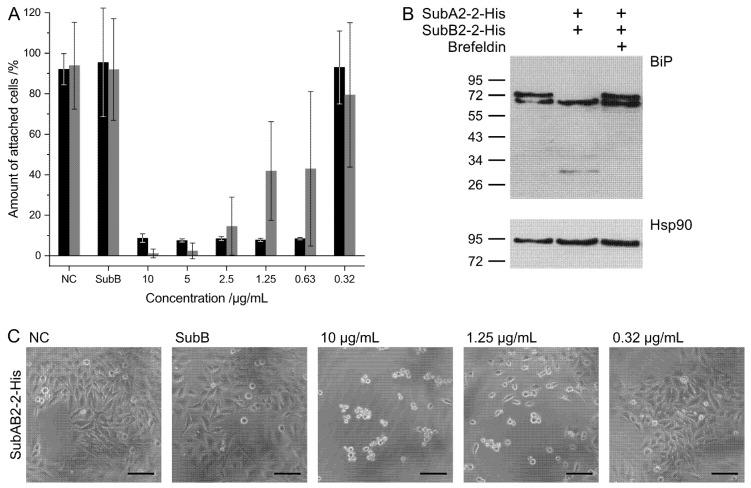
Cytotoxic effects of SubAB1-His (black) and SubAB2-2-His (gray) toward HeLa cells. (**A**) Cells were incubated at 37 °C with the indicated combinations and concentrations of the respective proteins in the medium. After 72 h of incubation, the amount of attached (non-intoxicated) cells was determined. The subunits were separately purified and mixed in a 1:5 molar ratio prior to the application to the cells. For control, the cells were left untreated (negative control, NC) or treated with SubB alone. The results are obtained by three independent experiments and shown are the average values from these experiments. (**B**) Western blot analysis of SubA2-2-catalyzed proteolytic cleavage of BiP in HeLa cells treated for 4 h at 37 °C with the indicated combination of SubA2-2-His and SubB2-2-His. In the right lane, 30-min pre-incubation with Brefeldin A was carried out. Cells were lysed, and equal amounts of proteins were subjected to SDS-PAGE and Western blotting with a specific antibody against BiP. The upper panel shows the non-cleaved and cleaved BiP. To confirm comparable protein loading and blot transfer of the proteins, the amount of Hsp90 was detected from the same samples (lower panel). (**C**) Analysis of the SubAB2-2-His-induced cell rounding. HeLa cells were incubated at 37 °C with increasing concentrations of the combination of SubAB2-2-His, with SubB2-2-His alone or left untreated (negative control, NC). Representative images of the cells are shown, reflecting the change in morphology in cells treated with SubAB2-2-His. The scale bar indicates 50 µm.

To ensure that the purified proteins were biologically active, the cytotoxicities of combinations of SubA-His and SubB-His subunits on HeLa cells were analyzed. Cells were incubated with SubAB1-His (black) or SubAB2-2-His (gray) combinations in the medium, and the amount of attached, i.e., non-intoxicated cells, was determined as described below. As controls, cells were left untreated (negative control, NC) or incubated with SubB1-His or SubB2-2-His alone. The SubAB1-His and SubAB2-2-His combinations exhibited the expected cytotoxic effects in a concentration-dependent manner, while SubB1-His and SubB2-2-His alone had no effects (Figure 2A). Comparison of the results of the two SubAB variants indicates that SubAB1-His exhibited a stronger cytotoxicity with only approximately 10% attached cells at a toxin concentration of 0.63 µg/mL compared to the effect observed for SubAB2-2-His. SubAB2-2-His cytotoxicity resulted in increasing amounts of non-intoxicated cells below a toxin concentration of 2.5 µg/mL.

Moreover, the specificity of the intoxication of HeLa cells with SubAB2-2-His was further characterized by the detection of BiP, the specific cellular target for SubAB in the endoplasmic reticulum of target cells. Figure 2B shows the cleavage of BiP after 4 h intoxication of HeLa cells with a 1:5 combination of SubA2-2-His and SubB2-2-His. Importantly, no BiP cleavage was observed when the SubAB2-2-His combination was applied to the living cells in the presence of Brefeldin A, an established inhibitor of the retrograde transport of internalized SubAB into the ER of cells. This result clearly indicates the specificity of the observed cytotoxic effects by SubAB2-2-His. Functionality of the mixed SubAB2-2-His subunits could also be shown by morphologic changes in cytotoxicity assay with HeLa cells (Figure 2C).

### 2.3. Biochemical Characterization of Separately Expressed and Purified SubAB Subunits

**Figure 3:**
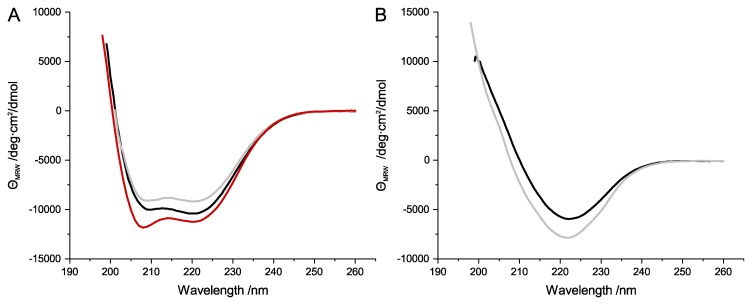
Far-ultraviolet (far-UV) spectra of SubA (**A**) and SubB subunits (**B**). (**A**) SubA1-His (black), SubA2-2-His (gray), and SubA2-2 (red) depicted typical α-helical signals in their far-UV spectra. (**B**) SubB1-His (black) and SubB2-2-His (gray) showed characteristic signals for proteins with high amounts of β-sheets.

Far-UV CD spectroscopy was used to investigate whether the purification protocols for SubA1-His, SubB1-His, SubA2-2-His, SubB2-2-His, and SubA2-2 resulted in folded subunits with comparable secondary structures. Samples of 200 µg/mL for each subunit were analyzed for their CD signal between 260 nm and 198 nm. Figure 3A,B shows a comparison of the obtained representative spectra. SubA1-His, SubA2-2-His, and SubA2-2 subunits revealed characteristic CD protein spectra with a high α-helix content (Figure 3A); α-helices in general show distinctive minima at 220 nm and 208 nm in the far-UV range [26]. The analyzed SubA subunits showed their maxima at exact the same positions.

In contrast, the SubB-His subunits (Figure 3B) showed CD signals which can be attributed to proteins with a high β-sheet amount in their secondary structure. β-sheets typically have their characteristic minimum and maximum at 222 nm and 190 nm, respectively [26]. Due to the limitation of the CD spectra, the maximum of the SubB-His signals were not measurable at 190 nm. However, the minimum at 222 nm with the subsequent increase of the CD signal with decreasing wavelength is a strong indication that the secondary structure of SubB1-His and SubB2-2-His contains β-sheets.

To analyze the thermal stability of the different subunits, thermal transitions were measured between 25 °C and 95 °C by CD spectroscopy. The loss of secondary structure was assessed at 220 nm for the A-subunits and at 222 nm for the SubB1-His and SubB2-2-His subunits. These wavelengths were chosen because they represent the wavelengths with the greatest change upon denaturation of the proteins. All SubA subunits showed an irreversible thermal denaturation with precipitation of the protein sample. This was also observed for SubB1-His and SubB2-2-His. The SubA-His subunits showed a clear single-step denaturation (Figure S1A,C,E, Supplementary Material). In contrast, the denaturation of the SubB-subunits appeared to be more gradual. This indicated that the structure of SubB undergoes small changes prior to ultimate denaturation (Figure S1B,D, Supplementary Material).

Simple logistic fits were used to determine the denaturation temperatures T_d_ of the subunits. For the A-subunits, denaturing temperatures of about 65 °C were calculated, where SubA1-His appeared to be slightly more stable with 66.5 °C in comparison to SubA2-2-His and SubA2-2 (Table 1). The latter subunits showed no differences in their *T_m_* with 64.7 ± 0.1 °C and 64.8 ± 0.1 °C. SubB1-His and SubB2-2-His revealed greater thermal stability in comparison to the A-subunits. In particular, the denaturation temperature of the B-subunits was detected to be approximately 80 °C. These results indicate that all analyzed A-subunits, as well as the B-subunits, are comparably folded (Table 1).

Oligomerization states of the various SubAB subunits were detected with size-exclusion chromatography on a Superdex200increase column running in standard measuring buffer (50 mmol/L Tris/HCl pH 7.5, 150 mmol/L NaCl, 10 mmol/L MgCl_2_). For SubA subunits, 6 µmol/L was analyzed, and, for SubB subunits, 30 µmol/L, and 6 µmol/L were analyzed. Two concentrations for the B-subunits were chosen to evaluate if the SEC results were concentration-dependent.

**Figure 4:**
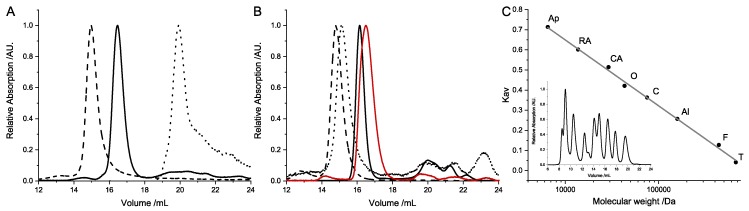
Representative normalized elution profiles of the recombinant SubAB subunits. (**A**) Data for SubAB1-His and (**B**) for SubAB2-2-His and SubA2-2. The A-subunits are represented by the solid curves; the corresponding B-subunits are the dashed curves. Denatured B-subunits are shown with dotted lines. All tagged subunits are colored in black and the untagged SubA2-2 is colored in red. The calibration curve for the calculation of the molecular weights is given in (**C**). The inlay in (C) shows a merge of the two calibration measurements. The following calibration proteins were used: thyroglobulin (T), ferritin (F), aldolase (Al), conalbumin (C), ovalbumin (O), carbonic anhydrase (CA), ribonuclease A (RA), and aprotinin (Ap).

All subunits eluted in a single sharp peak from the size-exclusion column (Figure 4A,B). This indicated one dominant species within the respective protein sample. The molecular weights of the subunits were calculated based on the calibration with the HMW and LMW kit from GE Healthcare (Table 2, Figure 4C). The molecular weights of SubA1-His, SubA2-2-His, and SubA2-2 were determined to be 25.3 ± 0.7 kDa, 25.8 ± 0.1 kDa, and 24.4 ± 0.5 kDa, respectively. These values were attributed to a monomer of the A-subunit. The elution volumes of SubB1-His and SubB2-2-His corresponded to 48.0 ± 2.0 kDa and 50.1 ± 0.1 kDa. Based on the theoretical molecular weights of the respective monomers, these results indicated the formation of homo-pentamers. No differences in the elution volume of the B-subunits were detected when measured at different concentrations (data not shown). This indicates that the detected pentamers were stable throughout the experiments performed subsequently.

### 2.4. Characterization of In Vitro SubAB Homo-Complexes

**Figure 5:**
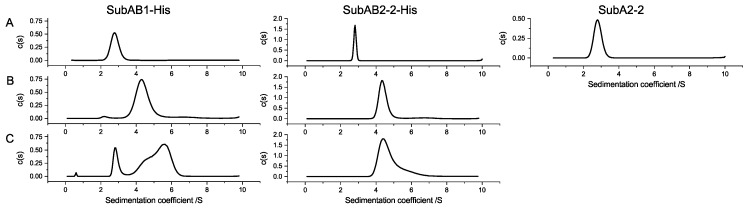
Results of the in vitro interaction of SubA and SubB analyzed with analytical ultracentrifugation. For each toxin variant, the subunits were analyzed separately ((**A**) A-subunits upper row, (**B**) B-subunits middle row) and as a mixture of both subunits (SubAB) in a molar ratio of 1:5 ((**C**) lower row). The analyzed variant is indicated by the header of each column.

The formation of homo-complexes was investigated with analytical ultracentrifugation. The centrifugation at 42,000 rpm of 6 µmol/L and 30 µmol/L samples of the variants of SubA1-His, SubB1-His, SubA2-2-His, SubB2-2-His, and SubA2-2, as well as the mixtures of SubAB1-His and SubAB2-2-His, were monitored with the absorption optic in an XL-A Optima centrifuge by Beckman. Data analyses were conducted with SEDFIT from Schuck [27]. Figure 5 depicts the results for the different SubAB variants. The single peak in the c(s) distribution of SubA1-His, SubB1-His, SubA2-2-His, SubB2-2-His, and SubA2-2 showed the sedimentation of one species. All SubA variants sedimented with a sedimentation coefficient of about 2.8 S, when measured alone. This corresponded to proteins of about 35 kDa in size. SubB1-His and SubB2-2-His both showed a sedimentation species with 4.3 S, indicating a molecular weight of about 65 kDa.

The left graph in the lower row in Figure 5 represents the sedimentation profile of a mixture of SubA1-His and SubB1-His. In the depicted curve, a distinct peak at ~3 S and a second peak at 5.6 S with a shoulder of about 4.5 S are visible, showing clearly that SubA1-His and SubB1-His formed holo-complexes in vitro with a sedimentation coefficient of 5.6 S. The separated peak and shoulder in this panel can be attributed to the remaining subunits, which did not form a complex at the given concentration. Analog measurement of SubAB2-2-His did not show comparable complex formation. The mixture of SubAB2-2-His resulted in a sedimentation species of 4.5 S with a clear tail toward higher S-values. In addition, no free SubA2-2-His was observable in this measurement, indicating that the A- and B-subunits of SubAB2-2-His only interacted transiently. Transient interactions are defined by fast association and dissociation [28]. This means that, for aUC experiments, the smaller species is entrained by the larger species, resulting in a single sedimentation peak with the S-value of the larger species, which then appears as a broadened or asymmetrical peak in the c(s) distribution [29].

**Figure 6:**
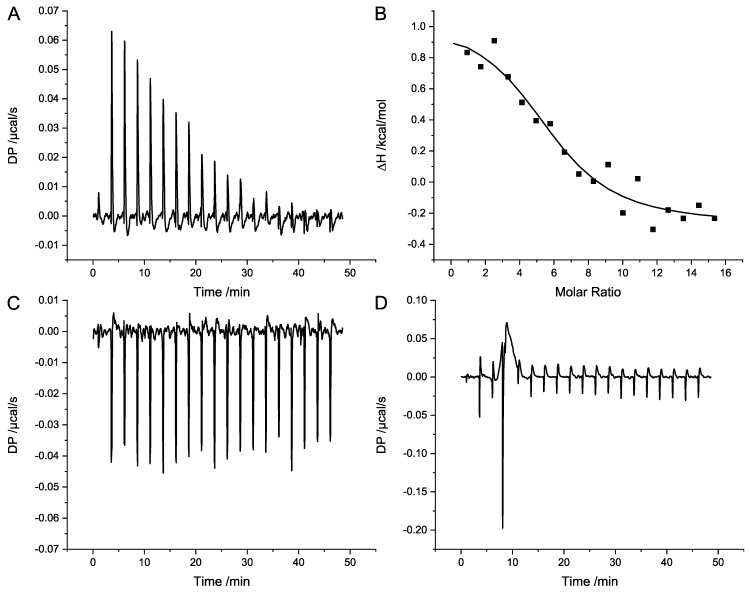
Isothermal titration calorimetry (ITC) analysis of the in vitro complex formation of SubAB. (**A**) Representative results of the endothermal complex formation of SubAB1-His; (**B**) corresponding analyses. Here, 320 µmol/L SubB1-His was titrated in 4 µmol/L SubA1-His. (**C**) Titration control of 320 µmol/L SubB1-His in buffer. (**D**) Titration of 320 µmol/L SubB2-2-His in 4 µmol/L SubA2-2-His.

For further characterization and the detection of the binding constant of the complex formation, isothermal titration calorimetry (ITC) was performed for SubAB1-His and SubAB2-2-His. For both variants, 4 µmol/L of the A-subunits were titrated with 320 µmol/L of the corresponding B-subunits in a MicroCal ITC device. Data analyses were performed with the original ITC analysis software from Malvern, applying a binding model with one set of sites. Figure 6A shows the titrations of SubAB1-His, revealing a clear endothermal binding signal for the complex formation. The heat uptake of the reaction is represented by positive signals in the titration. The integration and normalization to mol of injectant of the heat pulses is given in Figure 6B. The average of three independent titration resulted in a *K_d_* of 3.9 ± 0.8 µmol/L with a ΔH of 1.6 kcal/mol, −TΔS of −8.97 kcal/mol, and N of 4.9 ± 0.7. The titration control of SubB1-His is depicted in Figure 6C and shows that there was a constant thermal signal throughout the titration. The titration of 4 µmol/L SubA2-2-His with 320 µmol/L SubB2-2-His is shown in Figure 6D. No binding of SubB2-2-His to SubA2-2-His was detectable at these concentrations for this variant.

### 2.5. Binding of SubAB to HeLa Cells

**Figure 7:**
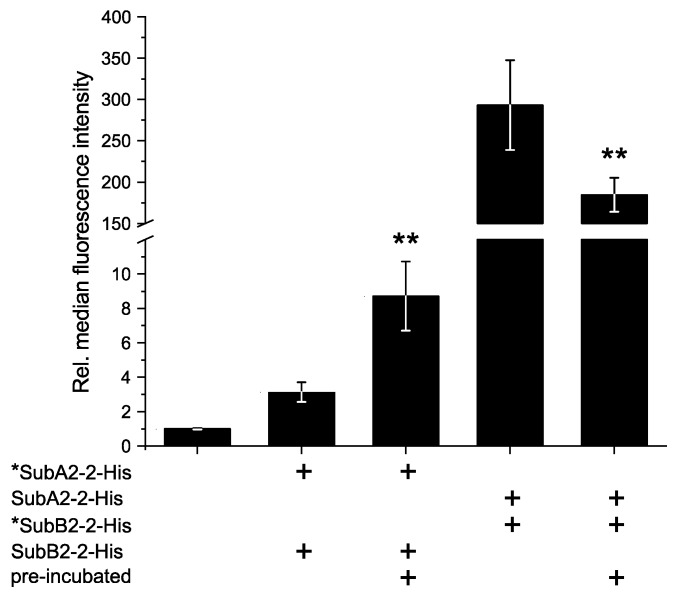
Flow cytometry (FACS) analysis of the surface binding of SubAB2-2-His to HeLa cells. Subunits were either pre-incubated prior to binding or directly applied to the cells. The asterisk * indicates the fluorescence-labeled component. Composition of each sample and the mode of application are indicated below the graph. The diagram shows the relative median of fluorescence intensity at 488 nm excitation. Values are normalized to the negative control (NC) and are given as mean of six technical replicates ± standard deviation (SD). SubA2-2-His and SubB2-2-His were mixed in 1:5 molar ratio, and 10 µg/mL or 5 µg/mL total protein concentration was used for *SubA2-2-His + SubB2-2-His or SubA2-2-His + *SubB2-2-His addition, respectively. Significance was tested using Student’s *t*-test; ** *p* < 0.01.

Flow cytometry experiments were performed to investigate the binding of SubAB complexes to HeLa cells and to elucidate whether pre-incubation of the toxin subunits compared to directly applied toxin subunits has an impact on the binding efficiency. Therefore, SubA2-2-His and SubB2-2-His were applied in a 1:5 molar ratio to simulate AB_5_ toxin stoichiometry. Figure 7 summarizes the result of the experiment with either DyLight 488 nm labeled A- or B-subunits. When labeled *SubA2-2-His and SubB2-2-His were applied directly, the relative median of the fluorescence signal was 3.1 ± 0.6, whereas a pre-incubation of these two toxin subunits resulted in a relative median of 8.7 ± 2.0. The results show that, due to the pre-incubation, the fluorescence signal of the A-component was approximately doubled. In contrast, a pre-incubation of *SubB2-2-His with unlabeled SubA2-2-His revealed a slightly reduced fluorescent signal (185.0 ± 21.0) compared to direct application of these two components (293.0 ± 54.0).

### 2.6. In Vitro Formation of Hetero-Complexes

**Figure 8:**
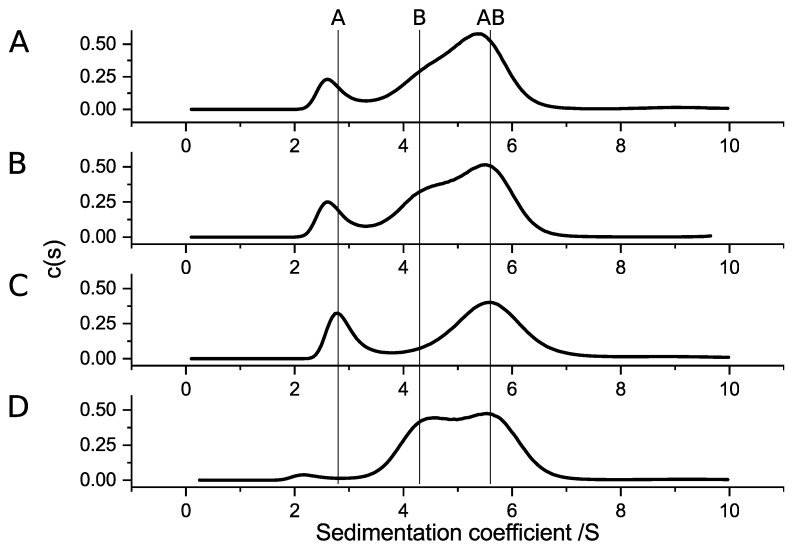
The c(s) distributions of hetero-complex formation of different SubA and SubB subunits. (**A**) Interaction of SubA1-His and SubB2-2-His; (**B**) SubA2-2-His interaction with SubB1-His; (**C**) c(s) distribution for SubA2-2 and SubB1-His; (**D**) result for SubA2-2 and SubB2-2-His. For all combinations, 6 µmol/L of the respective SubA subunits and 30 µmol/L of the respective SubB subunits were used. The vertical lines indicate the peak position of separated SubA1-His (A), SubB1-His (B), and the complex SubAB1-His (AB).

Hetero-complex formation of SubAB variants was analyzed with aUC by mixing 6 µmol/L of the A-subunits with 30 µmol/L of the B-subunits (Figure 8). Based on the results for the homo-complex formation, SubA1-His was mixed with SubB2-2-His (Figure 8A). SubA2-2-His was analyzed together with SubB1-His (Figure 8B), and SubA2-2 was analyzed together with SubB1-His (Figure 8C) and SubB2-2-His (Figure 8D). All analyzed combinations showed a clear maximum at about 5.6 S, which indicates that stable hetero-complexes were formed between the subunits of different origin. The sedimentation values, which were detected for the separated SubAB1-His subunit and the mixture, are indicated with vertical lines in Figure 8.

## 3. Discussion

In this study, we demonstrated, for the first time, that toxin complex formations of SubAB variants have different characteristics. Stable complexes not only assembled in solution, but most likely also on the surface of the target cells. Furthermore, our results indicate that a stable pentamer of B-subunits is mandatory for the AB_5_ complex formation in solution.

AB_5_ toxins are structurally characterized by an A-subunit which is predominantly composed of α-helices [30]. In contrast, the pentameric B-subunits typically contain a higher amount of β-sheets [31]. Crystal structure analyses of the functional SubAB holo-toxin complex by Le Nours et al. [32] showed that this fundamental distribution of secondary structure throughout the protein complex is true for SubAB1-His. In the current study, secondary structure analyses of the separately expressed and purified SubAB subunits revealed that SubA1-His, SubA2-2-His, and SubA2-2 showed typical α-helical signals in the far-UV CD spectra (Figure 3A). The His-tagged B-subunits also showed the expected pattern of proteins composed of β-sheets (Figure 3B).

SubA variants und SubB variants demonstrated different thermal stabilities. SubA subunits denatured irreversibly at approximately 65 °C, and SubB subunits denatured irreversibly at about 80 °C. The thermal stabilities indicated that the subunits used in this study were natively folded and stable at the temperatures that were applied throughout the experiments to characterize their complex formation capabilities. In addition, the His-tag had no observable effect on the secondary structure, thermal stability, and oligomerization of SubA2-2.

The separated aUC analyses of SubAB1-His subunits resulted in two distinct species, sedimenting with S-values which are comparable to the molecular sizes that were obtained from SEC analysis. This shows that SubA1-His is a monomer and SubB1-His a pentamer. The mixture of both subunits resulted in a new sedimentation species with an S-value and estimated molecular weight that correspond to the formation of an AB_5_ complex in solution (Figure 5 SubAB1-His, left column). The corresponding experiment for SubAB2-2-His showed comparable results for the single runs, but the results of the mixture of SubAB2-2-His did not reveal the formation of a peak with lager sedimentation coefficient, which would indicate an interaction species. Moreover, there was a clearly tailed peak observable at 4.8 S, which can be interpreted as the free B-subunits showing transient interactions with the A-subunit; however, no clear complex formation was observed for SubAB1-His (Figure 5 SubAB2-2-His, middle column) [29]. Possible reasons for the different complex-forming abilities could be the amino-acid composition of the variants. As mentioned earlier, the overall identity of the amino-acid sequence between the SubA and SubB subunits is 94.5% and 92.4%, respectively, which corresponds to 18 different amino acids in SubA2-2-His compared to SubA1-His and nine differences for SubB2-2-His compared to SubB1-His. No particular region was identifiable in the proteins where the changes in the sequence pile up. In contrast, the alterations were rather evenly distributed throughout the whole protein. In total, 13 amino acids throughout SubA1 were identified to be part of the interaction regions of the toxin complex [32]. None of them were different in SubA2-2-His. The same was true for the eight amino acids of the B-subunits, which were identified to be part of the interface between A- and B-subunits. However, in very close proximity to the AB interface, which is located between amino acids 91 and 94 in SubB, the proline at position 96 is altered to a leucine in SubB2-2 (amino-acid count includes the signal peptide). These two amino acids do not discriminate in their chemical properties but in their size. Based on this finding, we suspect that the missing ability of SubAB2-2-His to form stable toxin complexes in solution is caused by the leucine at position 96.

The analyses of complex formation of SubA1-His and SubB1-His with ITC revealed a dissociation constant in the lower micromolar range. This confirms the observation that this variant is able to form homo-complexes in solution. The corresponding experiments for SubAB2-2-His did not result in any binding signal given the applied condition, leading to the assumption that the SubAB2-2-His is not able to form stable complexes in solutions, or, if so, then only with a much larger dissociation constant. As described earlier, it is thought that AB_5_ toxins are assembled in complexes to be able to bind to their target cells and mediate their toxicity. Nevertheless, Pellino et al. [23] showed that the preassembly of the AB_5_ toxin complex is not necessary for intoxication by Stx. Furthermore, they showed that the assembly occurs at the receptor interface. Taking this new model for the functionality of AB_5_ toxins into account, our results led to the hypothesis that SubAB2-2-His forms a stable and functional toxin complex on the surface of the target cells.

To prove this suggestion, flow cytometry experiments were performed to understand the binding behavior of the separate SubAB2-2-His subunits. Pre-incubation resulted in a clear increase in the fluorescence for *SubA2-2-His + SubB2-2-His (Figure 7), which could be interpreted as better binding and could be attributed to the formation of AB_5_ complexes prior to the binding event. This is also supported by the observation made by the aUC experiments, which suggests the formation of transient SubAB2-2-His complexes (Figure 5 SubAB2-2-His, middle column). Contrarily, the results for SubA2-2-His + *SubB2-2-His showed that there is no positive observable effect on the binding. The observed reduction in fluorescence could be a consequence of the AB_5_ complex formation and binding to HeLa cells, as changes in the surrounding of fluorescent dyes tend to affect their signal [33,34]. In the cell-bound state of the complex, the fluorophores that are linked to *SubB2-2-His are sandwiched between the cell wall and the associated A-subunit. This constellation could shield the fluorophores and result in a decrease of the signal. Thus, this indicates that the formation of transient toxin complexes during the pre-incubation facilitates the binding to HeLa-cells and that stable SubAB2-2-His complexes can be formed at the target cell.

Furthermore, Funk et al. [24] showed, in cell culture-based assays, that SubAB subunits of different variants successfully intoxicated Vero cells. This suggests that the formation of hetero-complexes within the SubAB family is possible. Here, we investigated if the subunits of SubAB1-His, SubAB2-2-His, and SubA2-2 were able to form hetero-complexes (Figure 8). All combinations showed clear complex formation. Based on the results obtained in the previous experiments, we expected complex formation of all A-subunits with SubB1-His, because, so far, stable complexes were observed only for SubAB1-His. Furthermore, we assumed that SubB2-2-His is not able to form stable complexes in solution due to its amino-acid composition. However, Figure 8 shows different behaviors of the subunits. Specifically, while the complex formation of SubA2-2-His with SubB1-His and SubA2-2 with SubB1-His matched our expectations, this was not true for the complexes observed for SubA1-His with SubB2-2-His and for SubA2-2 with SubB2-2-His. Based on the size of the peaks corresponding to the AB_5_ hetero-complexes, samples containing SubB1-His as a B-subunit formed larger amounts of complexes compared to the mixtures with SubB2-2-His. In addition, SubA2-2 bound much better to the B-subunits (Figure 8C,D) than SubA2-2-His. This indicates that the His-tag linked to the toxin subunits has an effect on the biochemical properties.

Conclusively, we characterized the AB_5_ toxin complex assembly of different SubAB variants using aUC and ITC experiments. In our experimental settings, readily assembled B pentamers were mandatory for the complex formation. Furthermore, in comparison to other SubAB variants, SubAB1-His revealed the most stable complexes in solution. This is most likely attributed to the SubB1-His subunit, as other SubA variants also formed stable complexes with this subunit. SubAB2-2-His tended to form transient complexes in solution, which simplifies the binding to target cells. Nevertheless, stable complexes of this variant were not observed in solution, leading to our hypothesis that the cell surface is the main location of stable complex formation for SubAB2-2-His. However, further studies are required to fully understand the different properties of SubAB variants and their complex formations. In addition, it remains unclear how SubAB2-2 forms functional complexes which lead to a comparable intoxication of HeLa cells, as observed for SubAB2-2-His; thus, this requires further investigation.

## 4. Materials and Methods

### 4.1. Cloning, Expression, and Purification of SubAB Variant Subunits

#### 4.1.1. Cloning of SubA2-2

**Figure 9:**
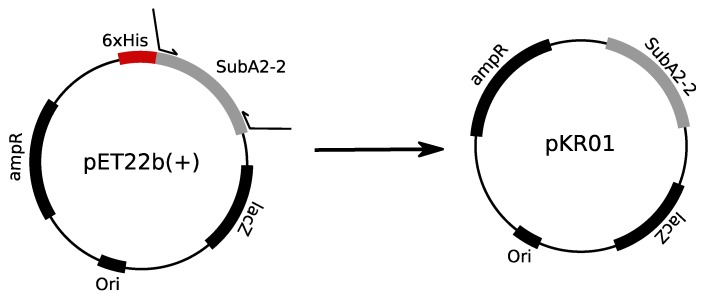
Schematic depiction of the cloning strategies for pKR01 starting from the pET22b(+) expression vector encoding SubA2-2-His subunit.

For expression of His-tag free SubA2-2, plasmids pKR01 was constructed. Therefore, the *subA* insert of the expression plasmids described by [24] were amplified in standard 50-µL PCR reactions using the Phusion High Fidelity Polymerase (Thermo Fisher Scientific, Waltham, MA, USA) with the primer pairs listed in Table 3 (Eurofins Scientific, Luxemburg, Luxemburg) according to the manufacturer’s manual to ensure that the sequences of the His-tagged and untagged subunits used in the previous and current study were identical. Prior to digestion with the restriction enzymes *Xba*I and *Xho*I (both Thermo Fisher Scientific, Waltham, MA, USA), the insert was purified with the Wizard^®^ SV Gel and PCR clean-up kit (Promega, Fitchburg, WI, USA) according to the manufacturer’s recommendations (Handbook 2009, Promega, Fitchburg, WI, USA). Digestion of the insert and the pET16b(+) vector (Novagen, Darmstaft, Germany) was performed as recommended by the web tool DoubleDigest calculator of Thermo Scientific. After inactivation of the restriction enzymes at 80 °C for 15 min, the insert was again purified with the Wizard^®^ SV Gel and PCR clean-up kit. The digested vector was loaded on a 1% (*w*/*v*) agarose gel, separated at 120 V with 90 min run time, and subsequently extracted with the Wizard^®^ SV Gel and PCR clean-up kit. Ligation was performed with a 1:5 molar ratio of vector to insert—applying 20 ng of vector—and the T4 Ligase (Thermo Fisher Scientific, Waltham, MA, USA) according to the manufacturers’ manual (Manual, 2014, Thermo Fisher Scientific, Waltham, MA, USA). The ligated DNA was transformed in chemical competent *E. coli* XL-1 Blue; positive clones were identified via colony PCR (using Primer pET16b_seq_for and pET16b_seq_rev, Table 3). A colony PCR was conducted in a total reaction volume of 20 µL containing 2 µL of template, 200 µmol/L dNTPs, 200 nM of each primer, 0.5 U of *Taq* polymerase, and 1× ThermoPol buffer. The templates were generated by suspending one colony in 20 µL of sterile water and incubation at 95 °C for 10 min of the cell suspension. Positive clones were verified by Sanger sequencing using the same primers as for the colony PCR after plasmid preparation (QIAprep Spin Mini Prep kit, Handbook, July 2006, Qiagen, Venlo, Netherlands). Sanger sequencing was performed with a CEQ™8000 automated sequencer (Beckman Coulter, Brea, CA, USA) as described previously [24]. The cloning strategy is depicted in Figure 9. Correct expression plasmids were transformed in *E. coli* C41 (DE3) to deliberately allow expression of the subunit.

#### 4.1.2. Expression and Cell Lysis of SubAB Variants

Expression of SubA2-2 subunit was performed in an auto-induction medium ZYM-5052 according to Studier [25]. The volume of a typical expression culture was 400 mL, which was composed of 200 mL of 2× ZY (2% (*w/v*) tryptone, 1% (*w/v*) yeast extract, pH 7.0), 8 mL of 50× M (1.25 mol/L Na_2_HPO_4_, 1.25 mol/L KH_2_PO_4_, 2.5 mol/L NH_4_Cl, 0.25 mol/L Na_2_SO_4_), 8 mL of 50× 5052 (2.5% (*w/v*) glycerol, 0.25% (*w/v*) d-(+)-glucose, 1.0% (*w/v*) lactose), 0.8 mL 1 mol/L MgSO_4_, and 150 µg/mL ampicillin. The expression culture was inoculated 1:50 with an overnight culture. Prior to inoculation, the overnight culture was pelleted (5000× *g*, 5 min, room temperature (RT)) and the cells were resuspended in fresh medium. Growth of the cultures took place at 37 °C with shaking at 180 rpm until an OD_600 nm_ of about 1.5 was reached and the auto-induced expression started. Subsequently, the culture was incubated for protein expression overnight at 20 °C and 180 rpm shaking. Expression was stopped by incubating on ice for 20 min. After that, the expression was harvested by centrifugation at 4000× *g*, 15 min, 4 °C. To remove residual medium components, cell pellets were washed in 30 mL of washing buffer (50 mmol/L Tris/HCl pH 7.5, 100 mmol/L NaCl) and then stored at −20 °C until purification. Expression of SubAB-His subunits was performed as described by [24].

Cell lysis of all expressions was performed in the first buffer using the subsequent purification. The cell pellets were resuspended in 5 mL of buffer/1 g cell pellet and supplemented with 1 mg/mL lysozyme. After an incubation of 1 h on ice, the cells were disrupted by sonication in 12 cycles with 10 s on time and 20 s off time, with an amplitude of 70% and a microtip at a Sonifier^®^ SFX150 Cell disruptor (Branson Ultrasonics Corporation, Danbury, CT, USA). The lysate was cleared by centrifugation at 30,000× *g*, 4 °C for 45 min and later used for purification.

#### 4.1.3. Purification of Recombinant Subtilase Subunits

SubA-His subunits were purified with gravity flow His-columns according to the protocol by Funk et al. [24]. For the removal of imidazole and remaining impurities of the sample, the SubA-His subunits were run on a Superdex75pg gel filtration column in standard PBS supplemented with 10% glycerol to increase the protein stability during storage (GE Healthcare, Chicago, IL, USA).

The SubA2-2 subunit was purified with a two-column approach on an ÄKTA pure system (GE Healthcare, Chicago, IL, USA) consisting of the cation exchange SPff column and a gel filtration Superdex75pg column (GE Healthcare Chicago, IL, USA). The cleared lysate was loaded via a 50-mL superloop on the first column equilibrated in SP1A buffer (50 mmol/L Tris/HCl pH 7.5, 5 mmol/L EDTA). After a washing step with five column volumes (CVs) of buffer A, elution was performed with a linear gradient against SP1 B buffer (50 mmol/L Tris/HCl pH 7.5, 5 mmol/L EDTA, 1 mol/L NaCl) and 2-mL fractionation. Protein-containing elution fractions were analyzed with 12.5% (*v/v*) SDS-PAGE (180 V, 35 mA per gel, 80 min), and all SubA2-2 positive fractions were pooled for polishing on a Superdex75pg column. Gel filtration was performed in standard PBS with 10% (*v/v*) glycerol. After identification by SDS-PAGE and pooling of the target protein-containing fraction, the pool was concentrated with Amicon Ultra-15 spin columns cut off at 10 kDa (Merck Milllipore, Burlington, MA, USA), aliquoted, and stored at −70 °C. Similar to the His-tagged A subunit, all His-tagged B-subunits were purified following the protocol by [24] with an additional gel filtration step on a Superdex75pg in PBS + 10% (*v/v*) glycerol.

#### 4.1.4. Concentration Detection

For all experiments, the concentration of the analyzed proteins was determined spectroscopically by measuring the absorption at 280 nm using a Nanodrop2000 device (Thermo Fisher Scientific, Waltham, MA, USA). The length of all subunits, molecular size, and corresponding extinction coefficient were calculated with ExPasy/Protparam and are given in Table 4.

### 4.2. Cytotoxicity Assay

The determination of the cytotoxicity of the respective SubAB complexes and separate subunits, assays with HeLa cells were performed as described elsewhere [24]. The detection of the assays was either performed as batch detection using crystal violet staining or direct counting of rounded and unaltered cells after 72 h of incubation with the applied toxins. For intoxication, the separate SubAB subunits were mixed in a 1:5 molar ratio, and total protein concentrations from 10 µg/mL to 0.32 µg/mL were transferred to the epithelial cells. This ratio was chosen to simulate the addition of AB_5_ holo-toxin complexes. For calculation of the average and standard deviation for cytotoxicity, three independent experiments (biological replicates) with three technical replicates each were performed.

### 4.3. Detection of BiP Cleavage

Verification of BiP cleavage in the intoxicated HeLa cells was performed with 1 × 10^6^ cells in 12.5 mL, seeded in 12-well plates distributed to 8 × 10^4^ cell per well. After overnight growth, the cells were washed one time with PBS and incubated with serum-free MEM or 10 µmol/L Brefeldin A (Selleckchem, Munich, Germany) in serum-free MEM for 30 min. Subsequently, the toxin was added, and the cells were further incubated for 4 h. In total, 10 µg/mL toxin was added to the cells containing the A- and B-subunit in a 1:5 molar ratio. After another washing step with PBS, cells were detached by adding 2.5× Laemmli sample buffer containing DTT and mechanically removed from the surface. Cells were transferred in reaction tubes to be incubated at 95 °C for 10 min. Then, 40 µL of each sample was applied to a 12.5% SDS PAGE. Following this, the proteins were blotted onto a nitrocellulose membrane, and BiP was detected using anti-GRP78/BiP rat (1:1000, Santa Cruz Biotechnology, Dallas, TX, USA) and anti-rat-HRP (1:2500, Cell Signaling Technology, Danvers, MA, USA). The detection was performed by enhanced chemiluminescence (ECL, Millipore, Burlington, MA, USA). For loading control, Hsp90 was detected after stripping of the blot using anti-Hsp90-mouse (1:1000, Santa Cruz, Dallas, TX, USA) and mBP (1:2500, Santa Cruz, Dallas, TX, USA).

### 4.4. Size-Exclusion Chromatography

Size-exclusion chromatography was performed in standard measurement buffer (50 mmol/L Tris/HCl pH 7.5, 150 mmol/L NaCl, 10 mmol/L MgCl_2_) on a Superdex200increase 10/30 GL column (GE Healthcare, Chicago, IL, USA). For an estimation of the molecular size and oligomerization of the separate subunits, the column was calibrated with blue dextran, aldolase, ovalbumin, conalbumin, carbonic anhydrase, and ribonuclease A (GE Healthcare HMW/LMW calibration kit, GE Healthcare, Chicago, IL, USA). The molecular weight was calculated by the determination of the *K_av_* value. All measurements were performed at least thrice.

### 4.5. Analytical Ultracentrifugation

Oligomerization and the formation of AB_5_ toxin complexes were analyzed by analytical ultracentrifugation (aUC). For each variant, the A- and B-subunits were analyzed independently and in a 1:5 molar mixtures to detect the formation of complexes. All measurements were performed in standard measurement buffer. The sedimentation of the samples was monitored in an Optima-XL-I Beckman centrifuge at 42,000 rpm with absorption spectra (Beckman Coulter, Brea, CA, USA). Data analysis was performed with SEDFIT v. 15.01b (2015) by Peter Schuck [27]. Viscosity and density of the solvent were calculated with SEDNTERP v.1.09 (2006) by Hayes, Laue, and Philo [35].

### 4.6. CD Spectroscopy

Experiments were performed at a Jasco J715 CD Spectrometer with a PTC-348 WI Peltier Unit using default settings (Jasco, Pfungstadt, Germany). Prior to the measurements, all samples were dialyzed in standard PBS buffer overnight. After centrifugation at maximal speed in an Eppendorf centrifuge, the concentration was detected with a Nanodrop2000 (Thermo Fisher Scientific, Waltham, MA, USA) and 200 µg/mL samples were prepared for measurement. Far-UV spectra were measured from 260 nm to 195 nm at 25 °C, and temperature transitions, monitored at 220 nm for the A-subunits and 228 nm and 218 nm for B-subunits, were recorded from 25 °C to 95 °C. Far-UV CD spectra were recorded with 100 nm/min of continuous scanning, 0.1 nm of data pitch, 4 s of response time, 1 nm of band width, and 10 accumulations. Raw data were recorded in millidegrees, and the signal was converted to the mean residue ellipticity (deg∙cm^2^/dmol). Temperature transitions were recorded with 1 °C/min of heating rate, 0.1 °C of data pitch, 1 s of response time, and 1 nm of band width. To evaluate the melting or denaturing temperature, all transition measurements were fitted with a simple logistic fit using OriginPro2019 from OriginLab (Northampton, MA, USA).

### 4.7. Isothermal Titration Calorimetry

To analyze the complex formation in vitro, isothermal titration experiments were performed using a Malvern PEAKCal device. All samples were prepared by dialysis overnight against the standard measuring buffer and concentrated with Amicon spinfilters (Merck Millipore, Burlington, MA, USA) to adjust concentrations. The standard assay settings were 750 rpm for stirring, with 5 µW used as the reference power; after 60 s of initial delay, the injection process started with 0.4 µL and an injection duration of 0.8 s. The first injection was followed by 18 injections with 2 µL and a duration of 4 s with 150 s of spacing. For all titrations, 300 µL of 4 µmol/L SubA was titrated with 20 µL of 320 µmol/L SubB solution in 19 steps at 25 °C. Evaluation of the titrations was performed with the MicroCal evaluation software from Malvern (Worcestershire, UK).

### 4.8. Flow Cytometry

To investigate cell surface binding, purified proteins were labeled with DyLight^TM^488 NHS ester (Thermo Fisher Scientific, Waltham, MA, USA) according to the manufacture’s protocol (Handbook 2011). After the labeling reaction, unbound dye was removed using Zeba Spin Desalting Columns (Thermo Fisher Scientific, Waltham, MA, USA). Activity of labeled components was verified via intoxication assays on HeLa cells and was comparable to the unlabeled variants. The labeled compounds were indicated by asterisk *.

HeLa cells were detached from culture plates using 25 mmol/L EDTA in PBS for 30 min at 37 °C and washed with PBS twice. Cells were aliquoted to 2 × 10^5^ cells/tube, and binding was performed with the indicated amount of labeled proteins for 2 min in PBS on ice. For the binding of pre-incubated toxins, the two protein subunits were pre-incubated together for 30 min in PBS on ice prior the addition to HeLa cells. After toxin binding, cells were washed with PBS once and subjected to flow cytometric analysis using the BD FACSCelesta (BD Biosciences, Franklin Lakes, NJ, USA). As a negative control, untreated Hela cells were used. Data were analyzed using Flowing software (version 2.5.1) by Perttu Terho.

## Figures and Tables

**Table 1:** Summary of denaturing temperatures of subtilase cytotoxins (SubA1-His, SubB1-His, SubA2-2-His, SubB2-2-His, and SubA2-2). † indicates that the temperature was estimated from the denaturing curve and not extracted form a logistic fit.

Sample	Denaturation Temperature (°C)
SubA1-His	66.5 ± 0.1
SubB1-His	80.0 ± 5.0 ^†^
SubA2-2-His	64.7 ± 0.1
SubB2-2-His	78.1 ± 5.3
SubA2-2	64.8 ± 0.1

**Table 2:** Molecular weights for SubAB subunits detected with size-exclusion chromatography (SEC) analyses in standard measuring buffer.

Subunits	Molecular Weight (kDa)
SubA1-His	25.3 ± 0.7
SubB1-His	48.0 ± 2.0
SubA2-2-His	25.8 ± 0.1
SubB2-2-His	50.1 ± 0.1
SubA2-2	24.4 ± 0.5

**Table 3:** Oligonucleotides used for the construction of expression plasmids pKR01.

Primer	Sequence	Constructed Plasmid
SubA2-2pET16b-for	CCT CTA GAA ATA ATT TTG TTT AAC TTT AAG AAG GAG ATA TAC CAT GCT TAA GAT TTT ATG GCC GCA	pKR01
SubA2-2pET16b-rev	GGG CTC GAG TTA CAG TTC TTC ACT CAT	pKR01
pET16b_seq_for	AGA TCT CGA TCC CGC GAA ATT AAT A	pKR01
pET16b_seq_rev	CCT CAA GAC CCG TTT AGA GGC	pKR01

**Table 4:** Overview of size, molecular weight, and extinction coefficient of all SubAB subunits used in this study. All sizes are without the signal peptide and were calculated with the online tool Protparam from ExPasy.

Subunit	No. of Amino Acids	Molecular Weight (kDa)	Extinction Coefficient (M/cm)
SubA1-His	334	36.1	41,035
SubA2-2-His	334	36.0	39,545
SubA2-2	328	35.2	39,544
SubB1-His	126	14.0	26,930
SubB2-2-His	125	14.0	26,930

## Supplementary Materials

The following are available online at https://www.mdpi.com/2072-6651/11/12/703/s1: Figure S1: Temperature transitions of SubAB subunits.
