# Supplementary material for: Variants of Escherichia coli Subtilase Cytotoxin Subunits Show Differences in Complex Formation In Vitro
Source: Toxins (Basel). 2019 Dec 3;11(12):703. doi: 10.3390/toxins11120703 (PMC6950094; doi:10.3390/toxins11120703)
Supplement: Supplementary file 1 [file toxins-11-00703-s001.pdf]

Supplementary Materials: Variants of *Escherichia coli* Subtilase Cytotoxin Subunits Show Differences in Complex Formation In Vitro

Maike Krause, Katharina Sessler, Anna Kaziales, Richard Grahl, Sabrina Noettger, Holger Barth, and Herbert Schmidt

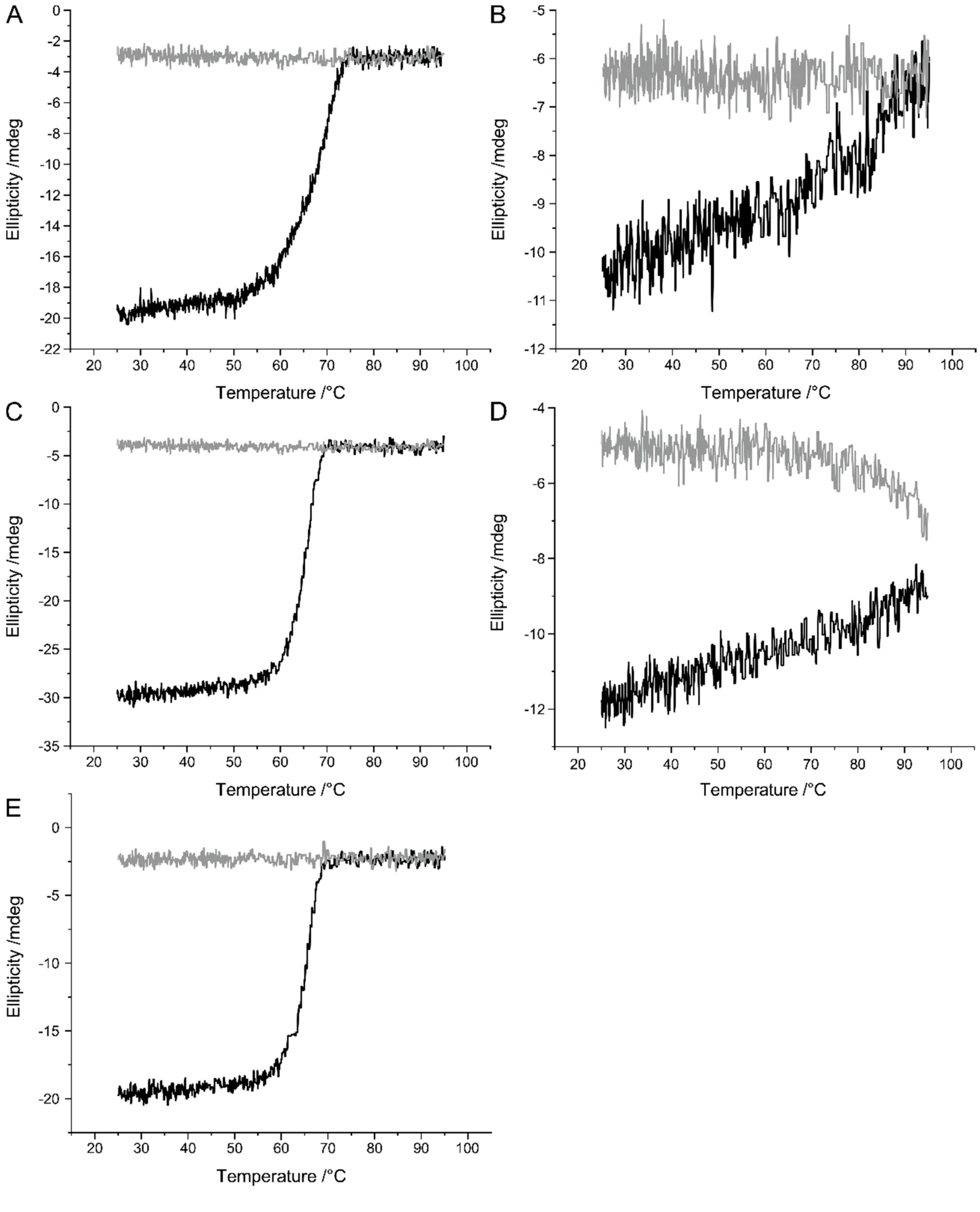

Figure S1. Temperature transitions of SubAB subunits. Depicted are the denaturing curve in black and the renaturing curve in gray. The transitions were measured at 220 nm for SubA1-His (**A**), SubA2-2-His (**C**), and SubA2-2 (**E**). SubB1-His (**B**) and SubB2-2-His (**D**) transitions were detected at 222 nm. All transitions were recorded from 25 °C to 95 °C and reverse after 180 s holding time.
